# Unsolved problems in CDH follow-up

**DOI:** 10.3389/fped.2022.977354

**Published:** 2022-10-26

**Authors:** Laura Valfré, Andrea Conforti, Francesco Morini, Neil Patel, Francesca Bevilacqua, Maria Chiara Cianci, Pietro Bagolan, Annabella Braguglia

**Affiliations:** ^1^Neonatal Surgery Unit, Medical and Surgical Department of Fetus-Newborn-Infant, Bambino Gesù Children's Hospital, IRCCS, Rome, Italy; ^2^Neonatal Surgery Unit, Meyer Children Hospital, Florence, Italy; ^3^Department of Neonatology, Royal Hospital for Children, Glasgow, United Kingdom; ^4^Unit of Clinical Psychology, Department of Neuroscience, Bambino Gesù Children's Hospital, IRCCS, Rome, Italy; ^5^Department of Systems Medicine, University of Tor Vergata, Rome, Italy; ^6^Neonatal Intermediate Care Unit and Follow-Up, Medical and Surgical Department of Fetus-Newborn-Infant, Bambino Gesù Children's Hospital, IRCCS, Rome, Italy

**Keywords:** congenital diaphragmatic hernia (CDH), pulmonary hypertension, neurodevelopment, hearing loss, thoracoscopy, problems, long-term follow-up

## Abstract

In patients affected by CDH, survival beyond the neonatal period continues to increase thanks to technological and pharmacological improvements. Conversely, patients, families and caregivers are more and more frequently facing “new” complex late comorbidities, including chronic pulmonary and cardiac dysfunctions, neurodevelopmental challenges, and specific nutritional requirements, that often require ongoing long-term medical or surgical care. Therefore, late morbidity is now a key focus in clinical care of CDH. The aims of this paper are to stress some of the most important “unsolved problems” for CDH patients at long-term follow-up.

## Introduction

For patients affected by severe CDH survival beyond the neonatal period is continuously improving due to technological and pharmacological improvements in care. A consequence of this are the complex constellation of unique comorbidities, including chronic pulmonary dysfunction, abnormal reactivity of the pulmonary vascular bed, neurodevelopmental challenges, hearing impairment, and nutritional challenges, became more and more frequent, and contributing to long-term medical and surgical care needs.

These longterm challenges may have an important impact on the quality of life (QoL) in CDH, which must be understood by clinicians who treat these children and their families (1).

Therefore, the worldwide focus of interest in CDH care is shifting to late morbidity; including the requirements for standardization of multicenter long-term follow-up programs, comparison of outcomes between centers, and evaluation of the long-term effects of interventions (2).

The aim of this paper is to stress some of the most compelling “unsolved problems” on CDH patients at long-term follow-up (LTFU).

## Prenatal assessments, fetal interventions, and long-term outcome

Prenatal unsolved problems focus:

- Prenatal predictors of long-term sequelae.
- Late outcomes of patients underwent prenatal intervention.
- Role of prenatal intervention on right sided CDH.

Prenatal diagnosis and advancements in prenatal and neonatal care have led to improved survival, but the risk of late morbidity remains high (3, 4).

The development and application of prenatal predictors aimed to estimate postnatal outcomes in CDH patients are well established (5). To date, the most widely used are the lung area to head circumference ratio (LHR), and the observed to expected LHR (O/E LHR) obtained using two-dimensional ultrasound (6, 7). Additional predictors have been proposed and investigated including mediastinal shift angle (5). These indices were initially developed with the goal of prenatally predicting postnatal CDH severity and its related mortality risk. With time, technological advancement, and increased standardization, this initial goal progressively shifted toward identification and selection of those most severely affected fetuses to offer fetal intervention (fetal tracheal occlusion, FETO), with the intention to modify post-natal outcomes. Recently, two multicentre randomized studies reported on the results of FETO in different severity-defined groups (8, 9). Despite the time and the efforts of these significant investigations, there is still uncertainty about real benefits of prenatal interventions, as highlighted by different authors (10).

In addition to these short-term two aims, a new ambitious goal has evolved: the ability to prenatally predict long term outcomes in CDH patients surviving the neonatal period.

However, to date, many controversies exist on the ability to prenatally predict long-term morbidity outcomes. In recent papers, prenatal risk stratification based on O/E LHR does not appear to predict a worse outcome in LTFU (11). Specifically, there is no clear association between a lower O/E LHR and a reduction in receptive expressive emergent language test, 3rd Ed. (REEL-3) or Bayley score, nor ventilation/perfusion (V/Q) mismatch. Neonates born with isolated CDH have similar measures of long-term morbidity, including neurological development and growth in height and weight, regardless of their O/E LHR (11).

Similarly, the impact of prenatal intervention on longterm follow-up remains unclear. Intrauterine tracheal occlusion appears to ameliorate and even reverse impaired lung growth in experimental models and in the human condition (8, 9). The technique appears to work by preventing the egress of liquid from the lung, increasing airway pressure, causing cellular proliferation, and increasing alveolar airspace and maturation of pulmonary vasculature.

One recently recognized complication of infants with CDH treated with FETO is tracheomegaly. Recently, McHugh et al. (12) and Zani et al. (13) reported cases of FETO-treated CDH fetuses presenting with respiratory distress shortly after birth, in whom marked tracheomegaly was identified, highlighting potential mechanical airway damage induced by *in utero* balloon occlusion.

Although FETO has a significant impact on tracheal size in CDH infants, the degree of tracheomegaly does not appear to impact survival or need for respiratory support in these infants. Further, the proportion of children with long-term respiratory infections appears to be similar between CDH survivors prenatally treated with FETO, and those who were not (12, 13).

Moreover, role of FETO in right sided CDH (RCDH) infants is poorly characterized: although a greater morbidity in RCDH infants is generally reported, similar mortality was reported in comparison to left CDH patients. Furthermore, considering fetoscopic procedures, in both left and right-sided CDH patients no significant differences in either mortality or short- or longer-term outcomes were reported (14).

## CDH, pulmonary hypertension and follow-up

Pulmonary hypertension (PH) unsolved problems focus:

- Natural history of PH beyond neonatal life
- Risk factors for PH at follow-up
- Plasma biomarkers to improve PH assessment
- Impact of new pre- and post-natal therapies

Pulmonary hypertension (PH) is a key component of disease pathophysiology in CDH. Excessive muscularisation and thickening of the pulmonary arterial vessels result in increased pulmonary vascular resistance and pulmonary artery pressure (PAP), and in turn to clinical instability by promoting hypoxic pulmonary-to-systemic shunting, and right (RV) and left (LV) dysfunction (15, 16).

CDH-related PH (CDH-PH) typically resolves in the first weeks of life, persistence beyond this time is associated with increased mortality, ongoing respiratory support and supplemental oxygen in the neonatal period (17–19).

There is limited understanding of the natural history and mechanisms of PH beyond neonatal discharge however, due to the rarity of CDH and challenges of PH assessment.

Longitudinal echocardiographic cohorts have demonstrated PH at discharge in 2–11% of cases, with a trend of ongoing resolution in the first 12 months (17, 20). However, cross sectional studies using echocardiography and cardiac catheterization have observed PH and RV dysfunction in the second decade of life in some CDH survivors (21–24).

Furthermore, up to 17% CDH cases are discharged on pulmonary vasodilator therapies (25). In the most severe cases chronic or progressive pulmonary vascular disease may contribute to functional restrictions and death in later life (26, 27). For all these reasons CDH-PH follow-up is therefore indicated to monitor PH resolution or progression, guide therapies, and minimize the potential impact on growth, development, functional status and survival.

No reliable risk factors for post-discharge PH have been identified to guide patient selection for follow-up. Wong et al. observed a correlation of fetal lung volumes and PH at 2–5 years, but no such relationship was observed by Fingeret et al. (28) and Wong et al. (29). Empirically, cases with clinical or echocardiographic evidence of PH at discharge or receiving pulmonary vasodilator or oxygen therapy should be routinely followed up from discharge until PH resolution (20).

PH follow-up should be a component of a standardized, multi-disciplinary service, including access to specialist cardiology/PH expertise, and with careful attention to associated factors including nutrition and gastro-oesphageal reflux (30). Assessment and treatment of PH should be in accordance with international guidelines (31–33). Additional investigation, including cardiac catheterization should be guided by cardiology and PH experts in the team.

Many unknowns remain in post-discharge CDH-PH. Prospective multi-center, multi-model studies are needed to understand the pathophysiological mechanisms, risk factors, explore the roles of ventricular function, MRI, and plasma biomarkers for improved assessment, and the impact of new pre- and post-natal therapies (34–36).

## Respiratory outcomes

Respiratory unsolved problems focus:

- Natural history of pulmonary function during the long term.
- Predictors of late pulmonary function status in CDH survivors.
- Standardized strategies to reduce late respiratory problems (including RSV immunization to physical activities).

A standardized, multidisciplinary approach to CDH patients is essential to optimize respiratory outcomes at early and late follow-up (37–40).

CDH survivors may present with variable degrees of pulmonary hypoplasia, most often manifesting as recurrent respiratory tract infections (RTI) and/or obstructive symptoms (wheezing/asthma) (41–43). In recent series, the prevalence of RTI in CDH survivors ranges from 10% to over 50%: with an increasing trend of RTI during childhood from 10% at 6 months of age to 23% at 24 months of age. However, there is no evidence of a direct correlation between CDH severity and risk of developing RTI (44).

A recent large retrospective cohort study in CDH survivors observed a progressive decline of average pulmonary function in comparison to normative population standards (9): those with more severe CDH (defined as those with larger type C and D diaphragmatic defects) are at higher risk of deteriorating pulmonary function tests and may benefit from early recognition and monitoring for possible complications. Oxygen requirement at initial hospital discharge also correlated with decreased force expiratory volume by an average of 8.0% (45).

CDH survivors reaching adolescence and early adulthood often present with obstructive pulmonary symptoms, confirmed at spirometry testing (46). Some authors have observed that obstructive respiratory patterns can be detected early in life among CDH survivors and may be used to predict late respiratory outcomes (47). Finally, correlations between late pulmonary obstructive symptoms, neonatal pulmonary hypoplasia, and neonatal pulmonary hypertension have been reported. These findings reflect the intimate relationship between alveolar growth and maturation of the pulmonary vascular bed, both reduced in surviving patients with CDH (22, 46, 47).

Nevertheless, there are no definitive means of stratifying the risk of late pulmonary dysfunction in CDH survivors. This has led to a lack of standardized interventional strategies to reduce late respiratory problems in these patients. This includes a lack of quality evidence in relation to rates of RSV bronchiolitis and appropriate use of palivizumab viral prophylaxis in CDH patients (48).

## Gastroesophageal reflux

Gastroesophageal reflux (GER) unsolved problems focus:

- Late consequences of GER in CDH survivors.
- Timing and type of investigations to define GER.
- Treatment options for GER (pharmacological and surgical).

Approximately 60% of congenital diaphragmatic hernia (CDH) survivors present with long term sequelae, including pulmonary, neurological, and gastrointestinal morbidity. One of the most frequently reported disorders is gastroesophageal reflux (GER), which can lead to complications such as esophagitis and Barret esophagus, worsen or contribute to pulmonary morbidity, and is related to failure to thrive (30, 49).

A meta-analysis on patients with CDH performed by Machancoses and collaborators reported an overall prevalence of gastroesophageal reflux disease (GERD) of 53% in neonates and 35% in infants older than 1 year; a surgical anti-reflux procedure was required in 8–21% of cases. This meta-analysis highlighted a variability in the reported incidence, maybe due to the diagnostic method used. Current follow-up protocols suggest investigating GER only in presence of symptoms, but it may be underdiagnosed in asymptomatic patients if systematic esophageal monitoring is not performed (50–52). Therefore, in relation to the possible consequences of GERD in CDH survivors, Morandi et al. warranted a close follow-up even for asymptomatic patients, but the right timing and type of investigations (endoscopy, pH-impedance monitoring) for asymptomatic patients still needs to be defined (52).

In CDH patients, different mechanisms may contribute to the pathogenesis of GER: esophagogastric junction (EGJ) alteration, weakness of the crura, shortening of the esophagus, abnormal enteric innervation, impaired peristalsis, intestinal malrotation and increased post-surgery abdominal pressure (50, 51, 53). Rayyan et al. hypothesized that EGJ alteration may result from the diaphragmatic defect itself and its surgical treatment (54). Investigating esophageal motility and EGJ function with high-resolution manometry and impedance in CDH patients with and without patch repair, they found that peristaltic motor patterns in patients with CDH were comparable to controls demonstrating that the neural innervation of the esophageal body is preserved. On the other hand, EGJ end-exhalation pressure and inhalation-exhalation pressure difference were lower in patients with CDH primary repaired, suggesting that the activity of the crural diaphragm is reduced and that patch repair tightens the EGJ increasing flow resistance (54).

An optimal management of GERD requires reliable predictors that allow early preventative measures. Different variables were investigated as predictive of GER, both prenatal and postnatal.

Verla observed that larger defects and intrathoracic stomach displacement on prenatal MRI were significantly associated with the diagnosis of GERD, but an intrathoracic liver was not. On the other hand, these variables were not associated with the need of anti-reflux surgery (55). Cordier et al. found that stomach position on prenatal ultrasound was independently associated with GER. A correlation with the duration of parenteral nutrition and the persistence of oral aversion at 2 year was also mentioned (56). Therefore, in addition to predicting overall CDH severity in terms of postnatal mortality, need for prosthetic patch repair and use of extracorporeal membrane oxygenation (ECMO), stomach grading classification is a promising prenatal imaging factor predicting the postnatal occurrence of GER (56).

Fetal endoscopic tracheal occlusion (FETO) was mentioned as a possible factor increasing the risk of GER occurrence, but retrospective multi-center studies performed by Cordier and Leva revealed that the procedure does not impact on global gastrointestinal morbidity at 2 years of age (56, 57).

Several post-natal factors are associated with an increased risk of GER, including right-sided CDH, management with non-conventional mechanical ventilation such as high frequency oscillatory ventilation, need for nitric oxide (NO) and ECMO, the need for patch closure and liver within the chest (55, 58). On multiple variable analysis, however, Diamond et colleagues demonstrated that only liver in the chest and patch repair were significant predictors of GER. Patch repair seemed to be as well an independent predictive of anti-reflux surgery for patient with left-sided CDH (30, 55, 58). On the other hand, Meier and coworkers found no correlation between the incidence of GER and anatomical variations or between the preoperative herniation of the stomach and GER symptoms (51).

Therefore, despite several promising predictors for GERD, both prenatal and postnatal, no definitive and evidence-based predictor exist so far to drive GERD prophylaxis with certainty.

Treatment of GERD is based on pharmacological management with proton pump inhibitors (PPI) (59) and is recommended in CDH survivors during the first year of life. Oral PPI administration, however, presents some issues in infants: considering challenge in oral intake, granules are often crushed with subsequent variable degree of systemic drug exposure and administering suspending formula by gastric tube may lead to tube blockage. To overcome these limitations, Bestebreurtje suggested rectal administration of omeprazole (1 mg/kg), demonstrating results comparable to oral dosing in terms of increasing intra-esophageal and gastric pH (60). This therefore provides a promising alternative administration route for CDH infants with pathological GERD, but further studies are needed to introduce this method in clinical practice.

Despite medical treatment, symptoms of GERD often do not improve, therefore different additional approaches are required including use of nasogastric tubes (in ~25% of patients), enteral access procedures (gastrostomy or jejunostomy) or anti-reflux surgery (in 6–25% of cases) (50, 58, 61). Nasogastric tubes often complicate the establishment of eventual oral feeding; thus, their use is recommended for a limited period only. Prieto et al. identified characteristics of neonates with CDH independently associated with enteral access procedures during their initial hospitalization: oxygen requirement at 30 days, chromosomal abnormalities, gastroesophageal reflux, major cardiac anomalies, ECMO requirement, liver herniation and increased defect size. Based on these variables the authors established a clinical scoring system which may be considered in counseling and clinical decision making to better predicting the need for enteral access (53).

For patient with intra-thoracic liver and who received patch repair, anti-reflux procedures seem to be the management of choice for GERD and they are most commonly performed in the year after CDH repair (62). Performing fundoplication at a later stage for recalcitrant symptoms is often difficult due to adhesions, the presence of a synthetic patch and abnormal positioning of the spleen and liver (61), thus some authors have suggested one-step procedure with CDH repair. Few studies have analyzed the impact of preventive fundoplication at the time of CDH repair, suggesting that the procedure is safe and effective in preventing GER and growth disorders in patients with the intermediate or severe anatomical form of CDH and appears to improve post-operative oral feeding (61–63). Conversely, in patients with milder CDH, this approach would appear to prove more challenge than any benefits justify (61–63). Additionally, Meier et al. reported that infants benefit from fundoplication at the time of CDH repair only within the first year of life, while later the difference in GERD symptoms is not statistically significant compared to patients who did not undergo “preventative” anti-reflux surgery (51). Therefore, while intriguing, the role of preventative anti-reflux surgery in CDH patients remains unresolved.

## CDH neurodevelopmental outcome

Neurodevelopmental unsolved problems focus:

- Domain and methods to assess neurodevelopmental outcomes.
- Risk factors for neurodevelopmental impairment.

Neurodevelopmental impairment is recognized to be one of the most important sequalae in children born with CDH. Nevertheless, studies had provided only general understanding about neurodevelopmental morbidity and report variable incidence rates. The majority of studies focused on the first 3 years of life, indicating that CDH survivors are at risk for cognitive and motor dysfunction in between 16 and 80% of cases (64). However, there is no consensus regarding the different domains tested, as well as the different methods to test these domains (e.g., time frame, definitions of severity delays, etc.). This variation in testing has prevented a clear definition of possible neurodevelopmental impairment, and its correlation with different potential risk factors.

Nevertheless, despite this ambiguity, many authors agree that gross motor skills domain is the most impaired and least likely to improve (2, 65). Intrathoracic liver position, preterm delivery, 5-min APGAR, prolonged supplemental oxygen requirement, the use of ECMO, prolonged hospitalization, periventricular leukomalacia, initial neuromuscular hypotonicity as well as presence of associated anomalies are the most frequently reported risk factors for late motor impairment (65, 66).

Similar uncertainty is present in studies in preschool and school age CDH survivors. Neurocognitive impairment has been described in percentages varying from 0 to 40% of children (67, 68), while motor abilities appear to remain the most commonly impaired, in particular fine motor coordination, motor planning, and visual processing. Moreover, CDH survivors seem to be at increased risk for developing emotionally reactive and pervasive developmental problems, and higher risk of autism (68, 69).

When considering LTFU, a significantly higher proportion of CDH survivors will not achieve a school degree in comparison with general population. However, among those able to achieve a school degree, school achievements, educational level, and socioeconomic perspective are similar to age and sex-matched healthy controls (70, 71).

Finally, it must be considered that advances in neonatal intensive care, use of extracorporeal membrane oxygenation, and fetal interventions while increasing the chance to survive neonatal period, may contribute to increased burden of late neurodevelopmental morbidities.

## Sensorineural hearing loss

SNHL unsolved problems focus:

- Risk factors for SNHL (early and late onset).
- Length of appropriate follow-up.

In patients with CDH, SNHL has been reported with a variable prevalence, ranging from 0 (72) to 100% (73). Earlier studies tend to present a higher prevalence of SNHL, Amoils et al. (74) report a prevalence of SNHL over 50% and Alenazi et al. (75) found SNHL in 7 out of 38 (18%) CDH survivors. Controversies exist on the impact of the diagnosis of CDH *per se* on the risk of SNHL development. In a study on 111 ECMO graduates, Fligor et al. reported a 26% overall prevalence of SNHL in neonates with severe respiratory distress and described CDH as an independent risk factor (76). Conversely, a more recent study of 136 ECMO survivors observed a prevalence of 9% of SNHL, irrespective of the underlying diagnosis (77). As far as the natural history is concerned, in CDH patients, SNHL tends to present as late-onset and progressive. Most studies with data from neonatal hearing screening, report normal findings (73, 74, 78–82). Therefore, the extreme variability in length of follow-up in available reports, precludes firm conclusions on the actual prevalence.

The most frequently reported factors associated with SNHL are ECMO treatment (74, 76, 83, 84), length of mechanical ventilation and/or stay in the NICU or in hospital (74, 79, 80, 84–86), need for inhaled nitric oxide (85), patch repair (74), and dose and duration of specified drugs: loop diuretics (74, 79, 83–85), aminoglycosides (76, 84, 85), and pancuronium bromide (79, 85). Overall, these factors suggest that the most critically ill CDH patients are at greatest risk. On the other hand, Alenazi et al. found no association between CDH disease severity and risk of developing SNHL (75), suggesting that congenital factors may contribute to its development in CDH patients. It is possible that patients with CDH may be congenitally predisposed to a higher sensitivity to risk factors for SNHL. Identifying definite factors that place CDH patients at high risk for SNHL will permit their modification and may aid prognostication.

## Thoracoscopic vs. laparotomic surgery and long-term outcome

Surgical unsolved problems focus:

- Role of minimally invasive surgery.
- Timing of surgical repair in ECMO patients.
- Late surgical sequelae (minimally invasive surgery and open surgery).

Optimal surgical repair of CDH is still highly debated. Minimally invasive surgeries (thoracoscopic and laparoscopic) and open laparotomic approaches were used mostly based on surgeons' beliefs and experiences.

Although the choice between surgical options is poorly evidence-based, there is wide agreement that surgery should be delayed until physiological stability has been achieved and should be performed in elective circumstances (87, 88). Nonetheless, examining ECMO patients, international debate is still ongoing on the uncertainty surrounding optimal timing of CDH repair in infants on ECMO (89): the CDH Euro Consortium admits possible advantage to surgical repair during ECMO, while the Canadian CDH Collaborative and Congenital Diaphragmatic Hernia Study Group (CDHSG) advise delaying surgery until after ECMO weaning (87, 88).

Generally, minimal invasive surgery (MIS) is used in more stable patients, while more severe infants (e.g., those requiring HFOV or ECMO) are treated by open surgical procedures.

No definitive answer has been achieved on optimal surgical treatment, when considering the wide range of surgery-related morbidities reported after CDH repair. These include postoperative small bowel obstruction, feeding difficulties (requiring gastrostomy or fundoplication), and diaphragmatic hernia recurrence (90).

In general, surgical morbidity is directly linked to the method of repair. The major and most frequently reported downside of MIS in CDH repair is the higher risk of recurrence (91–93), reported three- to four-fold higher with the MIS approach. However, there is ongoing no definitive answer on poorer surgical outcomes for MIS, with some recent studies reporting similar recurrence rate between MIS and open repair (94). Furthermore, some authors reported an inverse correlation between risk of recurrence and surgeon's experience, proposing MIS to be limited to high-volume centers and experienced surgeons (95). Finally, the other single risk factor associated with higher recurrence rate is the defect size: it has been suggested to limit MIS to the smallest defects, classified as A or B by the Congenital Diaphragmatic Hernia Study Group (CDHSG) Staging System (93).

Conversely, a large CDHSG data series reported a five-fold increased risk of postoperative adhesive bowel obstruction in open CDH repair, when compared to MIS repair (95), although MIS patients had significantly less severe CDH.

Other Authors reported that up to 20% of CDH survivors may require operative intervention for a small bowel obstruction, regardless of the type of initial surgery, and those patients at increased risk include those who required patch repair (96).

In conclusion, both MIS and open surgery appear to be associated with benefits and weaknesses with no definitive advantages of one over the other.

In conclusion, all the efforts made to improve early survival in CDH patients have progressively shifted substantial attention to late sequelae. Long-term evidence-based data are still lacking, mostly due to the paucity of prospective multicentre studies.

The main unsolved problems in CDH follow-up can be summarized into four main groups:

1. Identification of risk factors (either prenatal or early perinatal) for late pulmonary function, PH, GERD and SNHL.
2. Correlation between prenatal predictors of late outcomes.
3. Characterization of neurodevelopmental outcomes.
4. Optimization of surgical approaches based on patients' clinical characteristics and needed.

The development of different international study groups may help to fill these knowledge gaps, further refining the quality of care offered, and improving patients' longterm quality of life.

Therefore, a possible programme for the next 3–5 years should be focused on optimization LTFU programs by:

- Creating standardized follow-up schedules at different time points, utilizing defined testing, to limit variation between centers.
- Implementing a LTFU international registry.
- Further promoting international multi-center studies.
- Planning a consensus statement on transitional care for CDH patients to adulthood.

To date, there are different international multi-institutional groups focusing their attention on the different topics of the above-mentioned agenda. Specifically, CDH Study Group and CDH Euroconsortium are promoting collaborative studies, implementing treatment guidelines, and exploring new treatments opportunities to improve CDH survival and late quality of life. More recently the European Commission pushed forward the creation of the European Reference Networks (ERNs). The ERNs are virtual networks involving health care providers throughout Europe with the task is to foster discussion about rare or complex conditions and diseases that require highly specialized care and concentrated knowledge and resources. CDH, a recognized rare and complex disease, is included into the European Reference Network for rare Inherited and Congenital Anomalies (ERNICA). ERNICA is a network (lunched in March 2017) of expert multi-disciplinary healthcare professionals from specialized healthcare providers across Europe aiming to pool together disease-specific expertise, knowledge, and resources otherwise unachievable in a single country. ERNICA aims to reduce health inequalities across Europe, standardizing practices and making high-quality care, by disseminating information and resources to healthcare providers, patients and their families across Europe, regardless of where their geographical localization. To achieve these aims, ERNICA promotes virtual discussion on complex cases, promotes development of “standards of care” (including clinical guidelines and consensus statements), conduction of multi-center high-quality disease-specific research, while developing standardized outcomes measures and data collection.

## Data availability statement

The original contributions presented in the study are included in the article/supplementary material, further inquiries can be directed to the corresponding author.

## Author contributions

LV and AB conceptualized the study. Each author was in charge for his/her own section, writing, editing each chapter. All authors approved final version.

## Conflict of interest

The authors declare that the research was conducted in the absence of any commercial or financial relationships that could be construed as a potential conflict of interest.

## Publisher's note

All claims expressed in this article are solely those of the authors and do not necessarily represent those of their affiliated organizations, or those of the publisher, the editors and the reviewers. Any product that may be evaluated in this article, or claim that may be made by its manufacturer, is not guaranteed or endorsed by the publisher.
